# Clinicopathological and bacteriological studies on lamb bacterial enteritis and monitoring the oregano oil and vitamins A,D_3_,E effect on its treatment

**DOI:** 10.5455/javar.2021.h514

**Published:** 2021-06-23

**Authors:** Asmaa Abdallah Darwish, Marwa Fawzy, Wafaa Abd-Latif Osman, Eman A. El Ebissy

**Affiliations:** Department of animal and poultry health, animal and poultry division, Desert Research Center, Cairo, Egypt

**Keywords:** Bacterial lamb enteritis, clinicopahtological parameters, oregano oil extract 15%, vitamins A,D_3_,E, *16SrRNA*

## Abstract

**Objective:**

The objective of the study was to assess the effect of A,D_3_,E (I/M) and oregano oil extract 15% on some clinicopathological parameters during lamb bacterial enteritis treatment.

**Materials and Methods:**

Sixty Barki lambs, 20 apparently healthy (control group) and, 40 suffered from bacterial enteritis [enteric group (EG)], were subdivided into four treated groups (TGs): antibiotic group (AG), antibiotic + A,D_3_,E group (A + A,D_3_,E), antibiotic + oregano oil (AOG), and oregano group (OG). Fecal swabs were collected from EG then aseptically cultured, isolated, phenotypically identified, genotypically confirmed, and sequenced by PCR *16srRNA*. Paper disk diffusion test was used for estimation of oregano oil extract 15% antibacterial activity. After blood sample aspiration from all animals, they were clinicopathologically and statistically analyzed.

**Results:**

*Escherichia coli*, followed by *Salmonella* species and then *Klebsiella* species, was the main causative agents of lamb diarrhea and were susceptible to oregano oil extract 15%. A + A,D_3_,E and AOG showed significant (*p *< 0.05) enhancement of some clinicopathological parameters more than AG or OG. Matrix metalloproteinases (MMP-2 and MMP-9) and total antioxidant capacity (TAC), yielded area under the curve, sensitivity, negative predictive value as 1, 100% and 100% respectively, were determined in both EG and TGs.

**Conclusion:**

Oregano oil extract 15% has good antibacterial properties against enteric bacteria *in vitro* and *in vivo*. The combination between antibiotic and antioxidant vitamins or oregano plant extract of 15% has a good impact on some clinicopathological alterations in lamb bacterial enteritis treatment. TAC, MMP-9, and MMP-2 may be good markers for the disease and its treatment follow-up.

## Introduction

Bacterial enteritis is a serious problem that negatively affects Barki sheep breeding development. *Escherichia coli*, *Clostridium* species (spp.), *Salmonella* spp., and *Klebsiella* spp. are the major causative agents of sheep bacterial enteritis. *Escherichia coli* and *Salmonella* spp. are normal inhabitants in the animal gastrointestinal tract and under stress conditions they activate and cause diseases [1,2]. *Clostridium* and *Klebsiella* spp. are found in the animal environment and are transmitted to animals through contaminated food, water, and tools. The infection by these species usually results in high morbidities and mortalities, especially at young ages [3,4]. Low feed conversion rates are also noted in the recovered animals due to irreversible organ damage. Furthermore, they have zoonotic importance [5]. Therefore, there is a persistent need to develop our treatment programs against it.

Many researchers referred to oxidative stress’s critical role in different diseases’ pathogenesis and symptom exacerbation in the last few decades. Others attributed the slow recovery rates to oxidative stress, resulted from the diseases and antibiotic administration [6,7]. So, antioxidants pharmaceutical products were incorporated in different disease treatment programs [6-9]. In the case of bacterial enteritis, vitamin A (*β*-carotene), vitamin E (*α*-tocopherol), and D_3 _(cholecalciferol) are the most important. Because of their potent antioxidant action and the effect of vitamin A in reducing the frequency and duration of diarrhea, the protective effect of vitamin D_3_ against diarrhea side effects and the pronounced anti-inflammatory characteristics of vitamin E [6-9].

On the other hand, natural plant extracts such as oregano *plant *extract approved their efficacy in several disease treatments and partially minimized the oxidative stress related to the diseases or antibiotic administration [10-12]. *Oregano plant (Oregano vulgare) *is a perennial shrub that belongs to the mint family, and mainly grows in the Mediterranean region. Its extract was employed in traditional medicine to treat many cases such as cutaneous sores, aching muscles, asthma, cramping, diarrhea, indigestion, common colds, and boosting overall health. In addition, recent studies have suggested it as a food preservative due to its good flavor and its lethal effect against a wide range of food spoilage bacteria [13-16]. In the veterinary practice, oregano plant extracts markedly controlled the* Clostridium perfringens* diarrhea in boilers and enhanced intestinal mucosa healing [17]. It also achieved amazing results against some antibiotic-resistant bacterial species. The therapeutic benefits of oregano plant extract were fundamentally attributed to its rich content of carvacrol and thymol volatile oils [12].

Hence, this research aimed to isolate and identify bacterial species associated with bacterial enteritis in Barki lambs phenotypically and genotypically, focusing on some clinicopathological alterations related to their infection. To determine the antibacterial properties of oregano oil extract 15% against isolated bacteria *in vitro*, to compare between the effect of vitamins A, D_3_, and E (intramuscular injection) and oregano oil extract 15% on some clinicopathological parameters during the disease treatment. We also evaluated the importance of matrix metalloproteinases (MMP-2 and MMP-9) and total antioxidant capacity (TAC) in the disease diagnosis and prognosis and its treatment follow-up.

## Material and Methods

### Ethical approval

The ethical approval was taken from Animal Health Department (Approval No.9, March 2020), Desert Research Center, Ministry of Agriculture and Land Reclamation, Egypt. 

### Animals

Sixty Barki female lambs (4–6 months of age) were kept at the Sustainable Development Center for Matrouh Resources (SDCMR). Twenty healthy lambs (physiological values of body weights, temperature, pulse, respiration rates, normal appetite, good posture, clean, bright eyes, no nasal discharges or diarrhea) were considered a control group (CG). The remaining 40, which suffered from diarrhea (watery whitish-yellow or grayish), hyperthermia, abdominal pain, dullness, depression, off food, emaciation, and weakness, were the enteric group (EG); they were subdivided according to their treatment proposals into four treatment groups (TGs) as follows: 

Antibiotic group (AG): 10 lambs were treated with oxytetracycline L.A. Norbrook^® ^Company (I/M injection, 20 mg/kg) repeated after 48 h + New-diaclean Swede pharm^® ^Company (orally 1/2 sachet for 3 consecutive days). 

Antibiotic + A,D_3_,E group (A + A,D_3_,E): 10 lambs were treated with oxytetracycline L.A. Norbrook^® ^(I/M injection, 20 mg/kg) repeated after 48 h + New-diaclean Swede pharm^® ^Company (orally 1/2 sachet for 3 consecutive days) + A,D_3_,E Univet group^®^ (I/M injection, 1 cm/10 kg). 

Antibiotic + oregano oil (AOG): 10 lambs were treated with oxytetracycline L.A. Norbrook^® ^(I/M injection, 20 mg/kg) repeated after 48 h + oregano oil extract 15% conc. New Vet Care^® ^(orally 5 ml for 3 consecutive days). 

Oregano group (OG): 10 lambs were treated with oregano oil extract 15% conc. New Vet Care^® ^(orally 5 ml for 3 consecutive days). 

The manufacturing companies prescribed all the dosages mentioned above and methods of administration. 

### Bacteriological examination

Fecal swabs were collected from EG and aseptically inoculated onto two plates under aerobic and anaerobic conditions [18]. The pure obtained colonies were identified and biochemically confirmed according to Sayah *et al.* [19] for *E. coli* and the standard operating procedure (SOP) [20] for *Salmonella* species. While *Klebsiella* species were isolated by fecal sample cultivation on MacConkey agar (Difco, Sparks, Md.) plates and tested by Enterotube II test (BiomerieuxVitek, Inc.). For more confirmation, positive samples were genotypically identified and sequenced by PCR *16srRNA* using QIAmp DNA mini kit, Germany, for DNA extraction. The primers mentioned in Table 1 define the amplified DNA region.

Agarose gel electrophoresis was carried out according to Sambrook and Russell’s [21] method. All instructions of the manufacturers were followed carefully. The antibacterial activity of oregano oil extract 15% against the isolated bacterial strains was determined *in vitro* by paper disk diffusion method [22,23]

### Clinicopathological examination

Blood samples were collected via jugular vein puncture from CG one time at the beginning of the experiment. The blood samples were collected from EG before treatment and a week post-treatment. Each blood sample was split into two portions, EDTA was added to one portion to interfere with the coagulation process. This portion was instantly used for hematological parameters estimation [24], while the other portion was allowed to coagulate, and centrifugated at 3,000 rpm for 20 min at 37°C to get serum for biochemical parameters evaluation by spectrophotometer using Biodiagnostic company^®^ kits. MMPs levels in serum were determined using ELISA kits of Cloud-Clone Corp Company^®^ Huston, TX. All manual instructions were carefully taken into consideration.

**Table 1.** Primers used to identify the isolated bacterial strains in PCR *16srRNA*.

Gene	Forward	Reverse	Accession number	Amplicon (bp)
*E. coli*	AATTCCAGGTGTAGCGGTGA	TTTTAACCTTGCGGCCGTAC	NR_024570.1	235
*Klebsiella *	CTCATGCCATCAGATGTGCC	CTCATGCCATCAGATGTGCC	MW350091.1	483
*Salmonella *	TCGTTCATATTTGCCGGCTG	GGCTGACAAAGTCCTGGTTG	MW322036.1	231

**Table 2.** Bacterial isolates’ prevalence rates in EG.

No. of lambs affected with bacterial diarrheal infection	Isolated Bacteria			Biochemical test
40	*E. coli*	25	62.5%	Positive indole test
	*Salmonella *spp.	10	25%	Negative urease test
	*Klebsiella *spp.	5	12.5%	Examined by VITEK system

**Figure 1.**
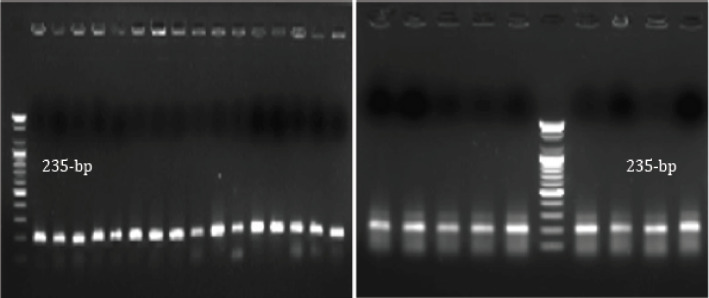
*16srRNA* for *E. coli*, positive results showed a band at 235 bp.

### Statistical analysis

Using SPSS^®^ version 24, one-way analysis of variance test was used to compare the estimated parameters’ means in the studied groups, and multiple comparisons Tukey’s HSD test was used to estimate the post-hoc differences between them (significant when *p *< 0.05). While GraphPad Prism version five5 program was used to compare the area under the curve (AUC), cut-off points, sensitivity%, specificity%, and likelihood ratio (LR) of MMPs and TAC in EG in relation to CG and TGs in relation to EG.

The positive predictive value (PPV), negative predictive value (NPV), and accuracy rate for them were estimated by the next equations: 

*PPV = True positive ÷ Total positive × *100.

*NPV = True negative *÷* Total negative *× 100.

*Accuracy rate = *(*True positive ÷ True negative*) ÷* Total population *× 100.

## Results

### Bacteriological results

**Figure 2.**
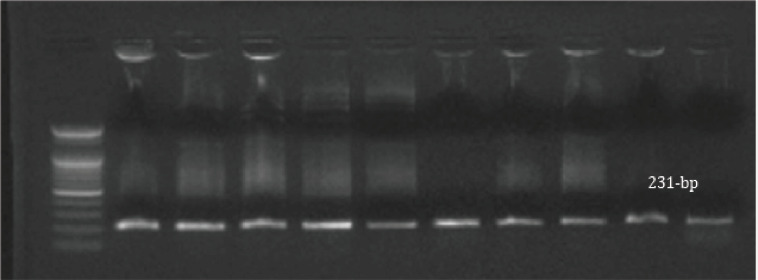
*16srRNA* for *Salmonella*, positive results showed a band at 231 bp.

**Figure 3.**
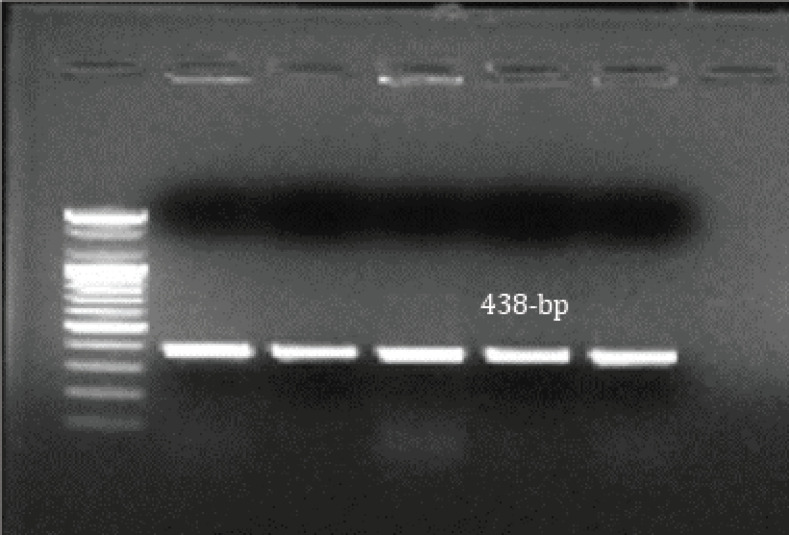
*16srRNA* for *Klebsiella*, positive results showed a band at 438 bp.

Out of 40 samples, 62.5% (25/40) had *E. coli*, 25% (10/40) had *Salmonella* species, and 12.5% (5/40) had *Klebsiella* species (Table 2). These isolates were identified genetically by *16S rRNA* PCR, whereas all *E. coli *tested isolates produced amplicon with molecular size 235 bp (Fig. 1). All *Salmonella* tested isolates produced amplicon with molecular size 231 bp (Fig. 2). All *Klebsiella* tested isolates produced amplicon with molecular size 438 bp (Fig. 3). 

### Paper disk diffusion test

Oregano oil 15% suppressed all isolated bacterial strains’ growth (which appeared as a transparent halo without growth around each disk). 

### Clinicopathological results

#### Biochemical results

Table 3 shows a significant (*p *< 0.05) elevation in serum levels of total protein (TP), albumin (Alb), globulin (Glob), glucose, kidney function tests [blood urea and creatinine (Cr)], liver enzymes [aspartate aminotransferase (AST), alanine aminotransferase (ALT), and alkaline phosphatase (ALP)], total lipids, triglycerides, MMP-2, and MMP-9 in EG when compared to CG. On the contrary, there was a significant (*p *< 0.05) depression in Alb/Globulin ratio (A/G), cholesterol, HDL-cholesterol (HDL-c), LDL-cholesterol (LDL-c), minerals (Ca, Mg, P), electrolytes (Na, K, Cl), trace elements (Zn, Cu), TAC in EG in relation to CG.

#### Hematological parameters

All red blood cell parameters and indices [red blood cell count (RBCs), hemoglobin concentration (Hb), packed cell volume (PCV), mean corpuscular volume (MCV), mean corpuscular hemoglobin (MCH), and mean corpuscular hemoglobin concentration (MCHC)], total leukocytic count (TLC) and neutrophils significantly (*p *< 0.05) increased in EG when compared to CG. Contrawise, lymphocytes displayed a significant (*p *< 0.05) decline in EG in relation to CG (Table 4).

#### Post-treatment

The four treatment protocols succeeded in stopping diarrhea, removing the other clinical symptoms, and most of the estimated clinicopathological parameters in TGs significantly (*p *< 0.05) went up or down till approaching CG levels [non-significantly (*p *≥ 0.05) changed with CG]. The comparison between the clinicopathological parameters of the four treated groups revealed only few differences. A + A,D_3_,E had the best and closest values to CG among the treated groups. It presented a significant (*p *< 0.05) improvement in TP, glucose, blood urea, Cl, K in AG, AOG, ALP, triglycerides, HDL-c, TAC, and Ca RBCs, Hb, MCH, neutrophils, and lymphocytes when compared to AG. AOG and AG ranked second and third, respectively, as AOG recorded a significant (*p *< 0.05) enhancement in triglycerides, cholesterol, HDL-c, P, RBCs, Hb, MCH, neutrophils, and lymphocytes’ numbers more than AG. Meanwhile, OG had the lowest degree of estimated parameters correction among TGs. As it demonstrated a significant (*p *< 0.05) increase in TP, Alb, glucose, Cr, ALP, total lipids, triglycerides, cholesterol, HDL-c, RBCs, Hb, PCV, TLC, andneutrophils related to AG, A + A,D_3_,E, and AOG, in AST and ALT related to A + A,D_3_,E and AOG, in Glob related to A + A,D_3_,E and blood urea associated with AG. Meanwhile, a significant (*p *< 0.05) decrease was detected between OG and other treated groups in LDL-c, Ca, P, lymphocytes and between OG and A + A,D_3_,E, AOG in TAC and Cl (Tables 3 and 4).

**Table 3.** Comparison between biochemical parameters in CG, EG, AG, AG + A,D_3_,E, AOG, and OG.

Parameter	CG (*n* = 20)	EG (*n* = 40)	TGs (*n* = 40)			
			AG (*n* = 10)	AG + A,D_3_,E (*n* = 10)	AOG (*n* = 10)	OG (*n* = 10)
Total protein (g/dl)	6.52 ± 0.24^f^	8.90 ± 0.06^a^	6.93 ± 0.02^b^	6.83 ± 0.02^b,c^	6.88 ± 0.05^b,d^	7.08 ± 0.04^b,c,d,e^
Alb (g/dl)	3.99 ± 0.20^f^	4.29 ± 0.06^a^	4.02 ± 0.07^b^	4.03 ± 0.07 ^b,c^	4.01 ± 0.07^b,d^	4.05 ± 0.03^b,c,d,e^
Glob (g/dl)	2.53 ± 0.30^f^	4.60 ± 0.09^a^	2.79 ± 0.06^b^	2.80 ± 0.06^b^	2.87 ± 0.08^b^	3.04 ± 0.05^b,d^
A\G	1.61 ± 0.27^f^	0.93 ± 0.03^a^	1.38 ± 0.05^b^	1.45 ± 0.05^b^	1.40 ± 0.06^b^	1.33 ± 0.03^b^
Glucose (mg/dl)	92.90 ± 2.07^f^	122.40 ± 1.71^a^	106.90 ± 0.88^b^	102.10 ± 1.66^b,c^	105.20 ± 2.66^b,d^	111.80 ± 1.75^b,c,d,e^
Blood urea (mg/dl)	24.74 ± 0.73^f^	44.68 ± 0.75^a^	27.22 ± 0.08^b^	26.36 ± 0.17^b,c^	26.98 ± 0.17^b,d^	28.77 ± 0.45^b,c^
Cr (mg/dl)	0.75 ± 0.11^f^	1.76 ± 0.11^a^	0.93 ± 0.02^b^	0.83 ± 0.06^b^	0.87 ± 0.02^b^	1.05 ± 0.03^b,c,d,e^
AST (U/l)	26.74 ± 1.61^f^	37.30 ± 1.19^a^	30.47 ± 0.21^b^	29.13 ± 0.60^b^	29.77 ± 0.40^b^	31.56 ± 0.19^b,d,e^
ALT (U/l)	36.74 ± 1.61^f^	47.28 ± 1.24^a^	39.48 ± 0.30^b^	38.73 ± 0.15^b^	39.01 ± 0.40^b^	40.73 ± 0.14^b,d,e^
ALP (U/l)	28.54 ± 0.30^f^	43.62 ± 1.37^a^	30.40 ± 0.12^b^	29.14 ± 0.04^b,c^	29.81 ± 0.11^b^	31.72 ± 0.63^b,c,d,e^
Total lipids (mg/dl)	355.40 ± 9.53^f^	373.45 ± 4.97^a^	354.44 ± 5.57^b^	346.67 ± 4.98^b^	348.13 ± 7.26^b^	363.87 ± 9.31^b,c,d,e^
Triglycerides (mg/dl)	73.17 ± 2.12^f^	136.05 ± 3.54^a^	87.48 ± 1.01^b^	79.83 ± 1.14^b,c^	82.03 ± 2.52^b,c^	92.01 ± 0.91^b,c,d,e^
Phospholipids (mg/dl)	161.04 ± 8.88	162.83 ± 1.94	156.51 ± 7.52	156.63 ± 7.05	155.32 ± 6.89	156.51 ± 7.52
Cholesterol (mg/dl)	121.19 ± 1.98^f^	74.58 ± 3.58^a^	110.45 ± 1.12^b^	110.23 ± 2.59^b^	110.78 ± 2.97^b,c^	115.36 ± 3.53^b,c,d,e^
HDL-c (mg/dl)	86.90 ± 1.39^f^	49.50 ± 2.22^a^	81.77 ± 0.83^b^	78.18 ± 1.17^b,c^	77.86 ± 2.67^b,c^	87.58 ± 0.81^b,c,d,e^
LDL-c (mg/dl)	34.29 ± 1.40^f^	25.08 ± 2.66^a^	28.68 ± 0.89^b^	32.05 ± 1.44^b^	32.92 ± 1.62^b^	27.78 ± 0.81^b,c,d,e^
Ca (mg/dl)	11.01 ± 0.26^f^	8.95 ± 0.03^a^	10.18 ± 0.07^b^	10.67 ± 0.48^b,c^	10.14 ± 0.27^b^	9.94 ± 0.07^b,c,d,e^
P (mg/dl)	6.35 ± 0.27^f^	4.88 ± 0.05^a^	5.75 ± 0.02^b^	5.97 ± 0.50^b^	6.06 ± 0.06^b,c^	5.05 ± 0.02^b,c,d,e^
Cl (mmol/l)	105.23 ± 2.64^f^	93.11 ± 1.68^a^	99.78 ± 0.45^b^	103.80 ± 1.52^b,c^	100.50 ± 0.34^b,d^	97.68 ± 1.80^b,d,e^
Na (mmol/l)	142.40 ± 2.80^f^	126.80 ± 3.41^a^	139.80 ± 1.75^b^	141.80 ± 1.75^b^	139.60 ± 0.84^b^	139.80 ± 1.75^b^
K (mmol/l)	3.47 ± 0.18^f^	2.91 ± 0.04^a^	3.05 ± 0.02^b^	3.20 ± 0.05^b,c^	3.06 ± 0.02^b,d^	3.05 ± 0.02^b^
Mg (mg/dl)	3.71 ± 0.50^f^	2.97 ± 0.04^a^	3.21 ± 0.39^b^	3.35 ± 0.48^b^	3.15 ± 0.32^b^	3.98 ± 0.04^b^
Cu (μmol/l)	23.55 ± 1.31^f^	20.62 ± 0.85^a^	23.70 ± 0.41^b^	23.84 ± 0.77^b^	23.76 ± 0.73^b^	23.62 ± 0.46^b^
Zn (μg/dl)	155.72 ± 7.65^f^	134.06 ± 6.37^a^	153.07 ± 7.36^b^	155.75 ± 7.31^b^	149.85 ± 2.85^b^	147.47 ± 1.88^b^
TAC (Mm/l)	1.23 ± 0.11^f^	0.71 ± 0.04^a^	0.84 ± 0.03^b^	0.95 ± 0.02^^b^,c^	0.94 ± 0.02^^b^,c^	0.84 ± 0.03^^b^,d,e^
MMP-2 (ng/ml)	15.39 ± 0.75^f^	65.49 ± 0.90^a^	16.96 ± 0.04^b^	16.70 ± 0.13^b^	16.66 ± 0.14^b^	16.96 ± 0.09^b^
MMP-9 (ng/ml)	22.75 ± 1.08^f^	71.89 ± 3.13^^a^^	24.27 ± 0.06^b^	23.57 ± 0.08^b^	23.95 ± 0.23^b^	24.48 ± 0.05^b^

Values are means ± SD.

a Significant with CG.

b Significant with EG.

c Significant with AG.

d Significant with AG + A,D_3_,E.

e Significant with AOG.

f Significant between studied groups, considered significant when *p* < 0.05.

Table 5 shows that MMP-2, MMP-9, TAC yielded AUC, sensitivity, NPV as 1, 100%, 100%, respectively, in both EG and TGs, but TAC achieved much better values of specificity, PPV, LR, accuracy rate than MMP-9 and MMP-2 in EG and TGs.

## Discussion

Bacterial diarrhea is an actual threat to sheep breeding. It widely attacks sheep flocks causing high mortalities, especially in lambs. Even after recovery, lower body weights and worse health are expected for the survived lambs [3,25].

In agreement with previous records [1,2], the traditional identification of isolated bacterial species by Gram-staining and biochemical methods revealed that the major bacterial cause of lamb enteritis was *E. coli*, followed by *Salmonella* spp. and *Klebsiella* spp. [26]. Their isolation percentages were 62.5%, 25%, and 12.5%, respectively. Lower or higher isolation rates may be reported before due to differences in the geographic area, feed and breeding system, age, and breeds. This result was more verified through *16SrRNA* gene analysis of isolated species, a gold standard for bacterial specification due to its high accuracy [27]. In order, *E. coli*, *Salmonella*, and* Klebsiella* spp. have been successfully sequenced with molecular size 235 bp, 231 bp, and 431 bp of *16SrRNA* gene.

**Table 4.** Comparison between hematological parameters in CG, EG, AG, AG + A,D_3_,E, AOG, and OG.

Parameter	CG (*n* = 20)	EG (*n* = 40)	TGs (*n* = 40)			
			AG (*n* = 10)	A + A,D_3_,E (*n* = 10)	AOG (*n* = 10)	OG (*n* = 10)
RBCs (×10^6^/μl)	12.59 ± 0.11^f^	14.11 ± 0.08^a^	12.94 ± 0.03^b^	12.70 ± 0.07^b,c^	12.77 ± 0.18^b,c^	13.53 ± 0.09^b,c,d,e^
Hb (g/dl)	14.18 ± 0.23^f^	16.91 ± 0.08^a^	14.86 ± 0.03^b^	14.29 ± 0.11^b,c^	14.44 ± 0.07^b,c^	15.12 ± 0.07^b,c,d,e^
PCV (%)	33.15 ± 0.81^f^	38.31 ± 0.24^a^	34.00 ± 1.49^b^	33.40 ± 1.17^b^	33.70 ± 1.06^b^	35.50 ± 1.27^b,c,d,e^
MCV (fl)	26.32 ± 0.62^f^	27.15 ± 0.23^a^	26.28 ± 1.19^b^	26.30 ± 0.86^b^	26.39 ± 0.96^b^	26.25 ± 1.94^b^
MCH (pg)	11.26 ± 0.23^f^	11.98 ± 0.09^a^	11.48 ± 0.04^b^	11.25 ± 0.12^b,c^	11.31 ± 0.20^b,c^	11.18 ± 0.08^b^
MCHC (%)	42.78 ± 1.08^f^	44.13 ± 0.32^a^	43.78 ± 1.92	42.84 ± 1.48^b^	42.88 ± 1.34^b^	42.65 ± 1.52^b^
TLC (×10^3^/μl)	7.21 ± 0.13^f^	11.19 ± 0.19^a^	7.78 ± 0.41^b^	7.81 ± 0.17b	7.85 ± 0.15^b^	8.23 ± 0.40^b,c,d,e^
N (×10^3^/μl)	2.15 ± 0.02^f^	7.14 ± 0.03^a^	3.15 ± 0.32^b^	2.95 ± 0.03^b,c^	2.96 ± 0.04^b,c^	3.75 ± 0.32^b,c,d,e^
L (×10^3^/μl)	4.06 ± 0.07^f^	3.09 ± 0.06^a^	3.65 ± 0.03^b^	3.89 ± 0.06^b,c^	3.92 ± 0.05^b,c^	3.50 ± 0.04^b,c,d,e^
M (×10^3^/μl)	0.44 ± 0.07	0.43 ± 0.07	0.41 ± 0.06	0.41 ± 0.06	0.41 ± 0.06	0.41 ± 0.06
E (×10^3^/μl)	0.53 ± 0.07	0.50 ± 0.09	0.52 ± 0.08	0.52 ± 0.08	0.52 ± 0.08	0.52 ± 0.08
B (×10^3^/μl)	0.04 ± 0.05	0.04 ± 0.04	0.04 ± 0.05	0.04 ± 0.05	0.04 ± 0.05	0.04 ± 0.05

Values are means ± SD.

a Significant with CG.

b Significant with EG.

c Significant with AG.

d Significant with AG + A,D_3_,E.

e Significant with AOG.

f Significant between studied groups, considered significant when *p* < 0.05.

**Table 5.** AUC, cut-off points, sensitivity%, specificity%, LR, PPV, NPV, and accuracy rate for MMP-2, MMP-9, and TAC in EG compared to CG and in TGs compared to EG.

	MMP-2 (ng/ml)		MMP-9 (ng/ml)		TAC (Mm/l)	
	EG	TGs	EG	TGs	EG	TGs
AUC	1	1	1	1	1	1
Cut-off points	15.9	64.97	23.70	71.44	1.06	0.78
Sensitivity %	100%	100%	100%	100%	100%	100%
Specificity %	70%	65%	75%	80%	95%	95%
LR	3.33	2.86	4	5	20	20
PPV	86.96%	41.67%	88.89%	83.33%	97.5%	95.24%
NPV	100%	100%	100%	100%	100%	100%
Accuracy rate	90%	72%	91.67%	90%	98.33%	97.5%

AUC = 0.86–1 (with satisfactory sensitivity and specificity: excellent marker)

LR < 5: low; = 5–10: moderate; >10: high.

These microorganism’s presence in the animal intestine, multiplication, migration, and accompanied tissue damage stimulates the pro-inflammatory cytokines to arrange an inflammatory immune response to destroy these pathogens [3,25]. They stimulate neutrophils production from the bone marrow [3,25]. Therefore, prominent neutrophilia and subsequent leukocytosis were observed in EG. They also encourage innate and humoral immune proteins genesis causing the detected hyperglobulinemia and the subordinate hyperproteinemia and decreased A/G ratio noted in EG [3,25]. In accordance with this hyperglobulinemia, a noted improvement in MMP-2 and MMP-9 activity was detected in EG in this work. MMPs play a critical role in immune response intensification by enhancing cellular migration and microbial phagocytosis [28,30]. This explains the lymphocytopenia reported in EG, as the lymphocytes migrated from the circulation to the intestine (site of infection) to destroy the bacterial invaders [3,4,25].

The activation of the pro-inflammatory cytokines leads to free radicals’ massive liberation from different immune cells. Under antioxidants control, these free radicals are assumed to react with the infectious agents to destruct them. In the present study, the free radicals exceeded the antioxidants neutralizing capacity and accumulated then reacted with different body cells causing their damage (kidney, liver, and intestine), and the oxidative stress appeared in EG (diminished TAC) [3,25]. Thus, oxidative stress, which started as a part of the host defense against the disease, is extensively incorporated in the raised hepatic and renal function tests and intestinal tissue damage detected in EG. Clinically, the pro-inflammatory cytokines are responsible for hyperthermia and pain sensation noted in the diseased animals via motivation of prostaglandin E2 secretion and bradykinin production [31,32].

The anorexia (due to hyperthermia and pain) damaged intestinal villi (due to oxidative stress) and the subsequent diminished absorptive surface had a major contribution in the distinguished hypocholesterolemia, HDL/LDL-hypocholesterolemia, decreased Ca, Mg, P, Na, K, Cl, Zn, and Cu serum levels in EG [3–5,25]. The demonstrated hypertriglyceridemia and the concomitant hyperlipidemia are other results for anorexia and intestinal tissue damage. The decreased fat absorption and lack of energy supplement enhance adipose tissue lipolysis and hepatneogensis, leading to intense triglycerides release and accumulation in the circulation. Therefore, the survived animals commonly suffer from weakness, emaciation, and poor body weight gain for a long time.

Reasonably, the excess fluid loss (due to intestinal epithelium damage) and related hypovolemia caused a pronounced hemoconcentration (represented in the current work by the significant increase in the erythrocyte parameters and indicies in EG), false hyperalbuminemia and hyperglycemia, raised liver and kidney function tests in EG [3,25]. These alterations translated clinically on the diseased animal by the lethargic look, dullness, and depression [3,25].

After treatment, the four groups markedly recovered, and diarrhea stopped owing to the bacterial elimination. Sequentially, the previous clinical symptoms disappeared and most of the clinicopathological parameters returned to their baseline values because of oxidative stress counteracting, intestinal absorptive surface repairing, and body fluids restoring.

Although the differences between the four treatment groups were not so considerable, the A + A,D_3_,E group had a remarkable improvement in some hematological parameters and biochemical parameters more than the other three treatment groups. Its values were nearer to CG levels. These findings were assigned to vitamin A magical effect on intestinal mucosa repairing and absorptive surface restoring [6,9], vitamin D_3 _antibacterial action [7], and vitamin E inhibitory action on the pro-inflammatory cytokines and related clinicopathological changes [8]. In addition, three vitamins are potent antioxidants; they worked on diminishing oxidative stress, which implicated in a lot of the clinicopathological alterations previously illustrated [6-9].

Similarly, the combination between the antibiotic and oregano plant extract 15% positively impacted some clinicopathological parameters in AOG compared to AG. As the oregano essential oils’ (carvacrol and thymol) antioxidant effect reversed, oxidative stress resulted from bacterial infection and/or antibiotic administration and subsequent alterations [10-12]. While their anti-inflammatory characteristics accelerated the inflammatory process termination and intestinal tissue healing [17].

Interestingly, the inhibition of bacterial growth in the paper disk diffusion test confirmed the oregano extract essential oils’ antibacterial action besides their antioxidants and antiinflammatory effects, particularly against *E. coli* (the highest bacterial isolate in the present data) [13-16]. This result was supported by the observed recovery of OG lambs and their clinicopathological parameters amelioration [10-12,17]. Rationally, the reported improvement of some clinicopathological parameters in A + A,D_3_,E and AOG more than AG or OG strongly recommended combining the antibiotic and antioxidant vitamins or oregano oil extract 15%.

The MMP-2, MMP-9, and TAC values of AUC, sensitivity, and NPV in EG and TGs suggested suitable markers for the disease diagnosis and prognosis and the treatment proposal tracking. While their specificity, LR, PPV, and accuracy rate numbers cleared, TAC was the best among them, followed by MMP-9 MMP-2.

## Conclusion

Oregano oil extract 15% has antibacterial characteristics against enteric bacteria *in vitro* and *in vivo*. A,D_3_,E injection or oregano plant extract is a valuable addition to bacterial enteritis treatment protocols. They are efficient in reversing the clinicopathological changes associated with the disease and reducing antibiotic side effects. TAC, MMP-9 and MMP-2 estimations may be noteworthy in lamb enteritis diagnosis and monitoring of its therapeutic programs wherein TAC is the best among them. It will be better if different oil concentrations were used and a larger number of animals were used. 

## List of Abbreviations

CG: control group; EG: enteric group; TGs: treated groups; AG: antibiotic group; A + A,D_3_,E: antibiotic + A,D_3_,E group; AOG: antibiotic + oregano oil group; OG: oregano group; TP: total protein; Alb: albumin; Glob: globulin; A/G: albumin/globulin ratio; AST: aspartate aminotransferase; ALT: alanine aminotransferase; ALP: alkaline phosphatase; Cr: creatinine; HDL-c: HDL-cholesterol; LDL-c: LDL-cholesterol; TAC: total antioxidant capacity; MMP-2: matrix metalloproteinase-2; MMP-9: matrix metalloproteinase-9; RBCs: red blood cell count; Hb: hemoglobin concentration; PCV: packed cell volume; MCH: mean corpuscular hemoglobin; MCHC: mean corpuscular hemoglobin concentration; TLC: total leukocytic count; N: neutrophils; L: lymphocytes; E: eosinophils; M: monocytes; B: basophils; PCR: Polymerase chain reaction; EDTA: ethylenediamine tetraacetic acid; HSD: honestly significant difference.
